# Circulation of the reassorted very virulent genotype of infectious bursal disease virus isolated from vaccinated broiler chickens in Bangladesh

**DOI:** 10.5455/javar.2024.k803

**Published:** 2024-08-20

**Authors:** Rony Ahmed, Md. Shamsul Kaunain Oli, Md. Salim Jahan, Sumaiya Pervin, Md. Mostakin Ahamed, Mohammad Habibur Rahman, Marzia Rahman, K. H. M Nazmul Hussain Nazir, Md. Tanvir Rahman, Md. Bahanur Rahman

**Affiliations:** Department of Microbiology and Hygiene, Faculty of Veterinary Science, Bangladesh Agricultural University, Mymensingh, BangladeshDepartment of Microbiology and Hygiene, Faculty of Veterinary Science, Bangladesh Agricultural University, Mymensingh, Bangladesh

**Keywords:** RT-PCR, very virulent infectious bursal disease virus, mutation, VP2 gene, phylogenetic analysis, amino acid

## Abstract

**Objective:**

The methodology employed in this research was designed to identify and characterize the infectious bursal disease virus (IBDV) at the molecular level, originating from recent outbreaks in Bangladesh.

**Materials and Methods:**

The IBDV outbreak farm was investigated, and bursa of fabricius (BF) specimens were acquired from infected chickens. Initially, viruses in the processed samples were detected in chicken embryo fibroblast (CEF) cells, and the RT-PCR method was used to confirm IBDV. The positive samples were injected through chorioallantoic membrane route into the embryo of a 10-day-old specific pathogen-free (SPF) egg for virus isolation and pathogenicity testing. Finally, we sequenced the VP2 gene to identify phylogenetic relationships and detect mutations.

**Results:**

From the 77 collected samples, 42.85% (33/77) were found positive for cytopathic effects in CEF cells, and IBDV was detected in 31.16% (24/77) of the samples by RT-PCR. IBDV was isolated in SPF chicken embryos. In the pathogenicity test, infectious bursal disease was evident in seronegative chickens with visible signs of disease. Sequence analysis shows that the broiler-isolated viruses clustered with genotype A3B2 and backyard chickens with genotype A1B1. The presence of amino acid motifs for virulence markers was revealed in the partially sequenced VP2 gene with a mutation at S254G in four IBDV isolates from broilers. However, amino acids for virulence markers were absent in two isolates from backyard chickens, which shows sequence homology with IBDV classic strains.

**Conclusion:**

In this study, we identified and characterized circulating reassorted IBDV from vaccinated broilers, which may be one of the major causes of vaccination failure in broilers.

## Introduction

The infectious bursal disease (IBD) virus causes an acute and contagious infection mainly in immature chickens that certainly attacks the bursa of fabricius (BF) and destroys B lymphocytes during their immature stage in the BF [1]. BF of infected chickens may appear edematous to hemorrhagic, swollen, and enlarged with characteristic yellowish transudate on the surface [2]. Early B cell damage results in compromised immunity in chickens, and they become more vulnerable to easy targets by other pathogenic and opportunistic organisms [1,3]. The severity of immunosuppression is subject to the virulence of the infecting virus strain and the age of the chicken. IBDV-infected macrophages may show reduced phagocytic ability [4,5], and lymphocyte cells in BF have been detected to be positive for IBD [1].

Two serotypes of this virus have been recognized: serotype-I and serotype-II, where serotype-I is associated with pathological conditions in juvenile chickens [6]. Serotype-I viruses are classified into attenuated (atIBDV), very virulent (vvIBDV), classical virulent strain (cvIBDV), and antigenic variant (avIBDV) viruses [7].

IBDV comprises two distinct segmented (Segment-A and Segment-B) two-stranded RNA (dsRNA), and genetic variation frequently occurs in the genome [3,8]. Hence, there is pathotypic variation, and antigenic and genetic diversity may occur due to the exchange of genetic material during pathogenesis or natural passage [9]. Due to the emergence of a novel IBD virulent virus, vaccine failure is occasionally reported on local farms, which is becoming a risk for the potential growth of the poultry industry. Again, variant types of IBDV serotype-1 emerge from antigenic variation, and vaccines manufactured from cvIBDV cannot protect against variant types [10,11]. Pathogenicity assessment of IBDV showed that segments A and B influence pathogenicity in chickens [12,13]. Genetic determinants for the pathogenicity of the virus are yet to be identified. However, mutations in the VP1 and hypervariable region (HVR) of the outer capsid of the VP2 gene influence the pathogenicity [14]. Analyzing the protein sequence of the VP2 gene, genetic markers for the virulence of the IBD virus were identified as amino acids at 253Q and 284A, where amino acids at 253H and 284T represent the vaccine strain [14]. Essential amino acids in VP2 were previously recognized in vvIBDV at positions 222A, 242I, 256I, 294I, and 299S [14]. This investigation was conducted for the isolation and characterization of IBDV from backyard chickens and vaccinated broilers. Phylogenetic and amino acid analyses were done to evaluate the relationship of the isolated IBDV with classic strains, vaccine stains, and variant strains.

## Material and Methods

### Ethical approval

The research involves the collection of samples of the BF from diseased chickens (field birds) and conducting a pathogenicity study following established protocols, with all laboratory procedures being carried out at the Virology Laboratory, Department of Microbiology and Hygiene, Bangladesh Agricultural University (BAU), Mymensingh 2202, Bangladesh. The utilization of live chickens and cell cultures in the laboratory experiment had been sanctioned by the Institutional Ethical Committee [approval number AWEEC/BAU/2018 (33)]. Moreover, before collecting the specimens, consent was sought from the owner of the flock.

### Sample collection

IBD-suspect BF specimens were pooled from infected chickens after primary investigation in three outbreak areas of Mymensingh, Gazipur, and Tangail in Bangladesh. Overall, 77 BF samples were randomly collected in virus transport medium (VTM) from vaccinated broiler (63) and backyard (14) chickens in sterile falcon tubes and transported via ice box in the Virology Laboratory of the Microbiology and Hygiene Department at Bangladesh Agricultural University.

### Sample processing and inoculum preparation

The collected samples were minced into small pieces and triturated to make a 20% suspension with phosphate-buffered saline (PBS) buffer. Samples were subjected to centrifugation at a speed of 6,000 rpm for 10 min at 4°C. The supernatant was separated, filtered through 0.22 μ syringe filters to remove bacterial and fungal contamination, and preserved at −80°C.

### Molecular detection of IBDV

According to instructions in the manufacturer’s manual, RNA was extracted from suspension utilizing a viral RNA separation kit, QIAamp (QIAGEN, USA), and RT-PCR was accomplished through the QIAGEN RT-PCR kit. Primers vv-fp775 (5´-AAT TCT CAT CAC AGT ACC AAG-3´) and vv-rp1028 (5´-GCT GGT TGG AAT CAC AAT-3´) were considered to bind the RNA sequence of the VP2 gene, encompassing alanine at 222 to isoleucine at 294 of the IBD virus [15]. Reverse transcription for complementary deoxyribonucleic acid (Cdna) synthesis was done at 42°C for 45 min, enzyme inactivation at 95°C for 5 min, and amplification of the cDNA cycle involved; 30 sec of fragment denaturation at 94°C, 1 min for primer binding at 45°C, prolongation of the fragment at 60°C for 1 min, and final elongation at 60°C for 10 min. The second primer (forward: 5´-GCC CAG AGT CTA CAC CAT-3´, reverse: 5´-CCC GGA TTA TGT CTT TGA-3´) was directed to multiply a 743 bp RNA portion of the VP2 gene from 710-bp to 1444-bp [16]. cDNA synthesis was done at 42°C for 1 h and the cDNA amplification cycle was as follows: 2 min of enzyme inactivation at 94°C, for PCR cycles, 1.5 min of DNA unwinding at 94°C, at 53°C for 1 min to anneal primer, allow prolongation for 1 min at 72°C, and an additional 5 min as final prolongation. PCR fragments were imaged under a UV-photometer after separating according to their molecular size with 2% agarose in Tris-Acetate-EDTA buffer (pH 8.0). IBDV live vaccine (GumboMed Plus Vet) virus, Incepta Vaccine Ltd., Bangladesh, was referred to as a PCR-positive control for each identification.

### Sequencing and evolutionary relationships

To identify the genotype, RT-PCR products of 254-bp and 743-bp of the VP2 gene were sequenced. Among 24 positive virus isolates, the VP2 gene of six isolates was partially sequenced by a third-party commercial service, “Invent Technologist Ltd., Bangladesh.” Nucleotide sequences were obtained from raw data using codon code aligner (Version 9.0.1) software, which was aligned with different strains of IBDV and considered for phylogenetic investigation by the maximum likelihood method (MEGA X software). Sequences were confirmed using BLAST (NCBI GenBank) online tools. The maximum likelihood approach, in combination with the Tamura-Nei model, was utilized to generate a phylogenetic tree to analyze the evolutionary association among various virus isolates. The Maximum Composite Likelihood scheme was considered to measure evolutionary distance, and units were described as per site-base substitution. Amino acids were inferred from the nucleotide sequences by using NCBI online software and compared with existing gene data in the NCBI GenBank using CLC sequence viewer 8.0 (QIAGEN).

### Virus isolation and harvesting

The IBDV was isolated in a 10-day-old SPF egg by injecting 0.2 μl of inoculum through the chorioallantoic membrane (CAM) approach and then placed in an incubator at 37°C. Embryo viability was routinely checked for 2–5 days after inoculation, and embryos with no viability after 24 h–5 days were cooled at 4°C for harvesting and processed to identify the virus.

### Pathogenicity of IBDV in seronegative chickens and re-isolation

Selected two IBDV isolates, BAU-MH-IBDV-BR-3 (test group BR-3) and BAU-MH-IBDV-BR-5 (test group BR-5) were used for the pathogenicity test in seronegative chickens [17]. For the test, three test groups were formed with chickens of 3 weeks of age: 8 chickens for each of the two isolates and a control group. A suspension of IBD virus (10^4.0^ egg infection dose 50/ml) from each isolate was administered ocularly to the test groups, and PBS to the control chicken. Test groups were kept separated to avoid horizontal transmission of the virus. The test groups were monitored for a period ranging from 3 to 10 days, and chickens that showed any disease manifestations of IBD were euthanized for observation; the BF and hemorrhagic muscles of infected chickens were processed for the detection of IBDV.

For further examination, histopathological analysis was carried out with the hemorrhagic portion of muscles and breast, kidney, and BF from the test chicken. Samples were fixed in 10% formalin buffer, followed by infiltration and implantation in paraffin. Sectioned (5 mm thick) paraffin-embedded blocks were stained utilizing hematoxylin and eosin Y, conforming to guideline procedures. Pathological alternations were investigated through microscopic study.

## Results

During this study, 3,250 chickens were visually inspected on the outbreak farms. High mortality and morbidity in broilers were observed compared to backyard chickens due to the IBD.

### Detection of IBDV in chicken embryo fibroblast cell lines designated CEF-1

Virus infection was detected in 33 (42.85%) samples with the visible identification of cytopathic effects (CPE) on the chicken embryo fibroblast cell (CEF-1) line. The infection was characterized as diminutive, circular, and reflective cells that separated from the flask wall and floated in the medium.

### Reverse transcriptase polymerase chain reaction result

**Figure 1.**
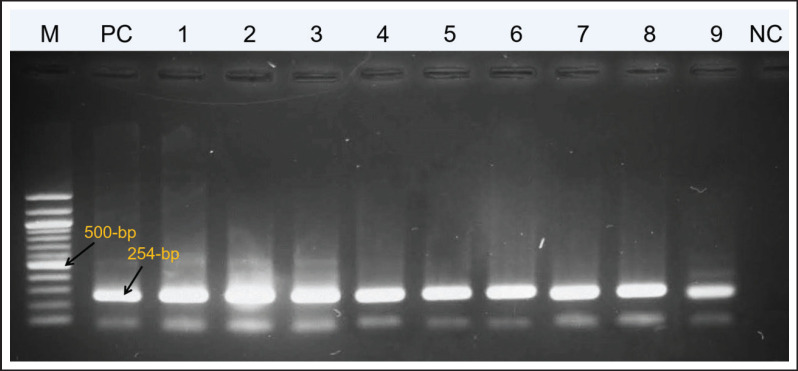
RT-PCR result of IBDV positive virus field samples. The positive control used in RT-PCR is the Incepta attenuated vaccine strain.

Among the 77 field-virus pathological identifications, 31.16% (24/77) of the specimens were found positive for IBD in RT-PCR identifications. Among them, 21 samples for broiler chickens and 3 for backyard chickens were found positive for IBDV in the samples (Fig. 1).

### Results of sequencing to identify reassortant IBDV strains and evolutionary relationships

The VP2 genes of six selected IBDV isolates were partially sequenced for molecular characterization and genogroup detection. Analyzing the raw nucleotide sequence data of 254-bp and 743-bp, a comparison of nucleotide and amino acid sequences was made with the VP2 gene of vvIBDV, cvIBDV, and the vaccine strain of IBDV. The sequences have been deposited in the GenBank database under the accession number provided in Table 1. Evolutionary analysis shows that four isolates from broilers are closely related to vvIBDV, and isolates from backyard chickens have genetic similarities with cvIBDV (Fig. 3). The phylogenetic tree constructed (Fig. 3) shows that the VP2 gene of broiler IBDV isolates shares the same clade as vvIBDV isolates, which belongs to genotype A3B2, while the VP2 gene of backyard IBDV isolates appears in the same clade as cvIBDV and belongs to genotype A1B1.

**Figure 2.**
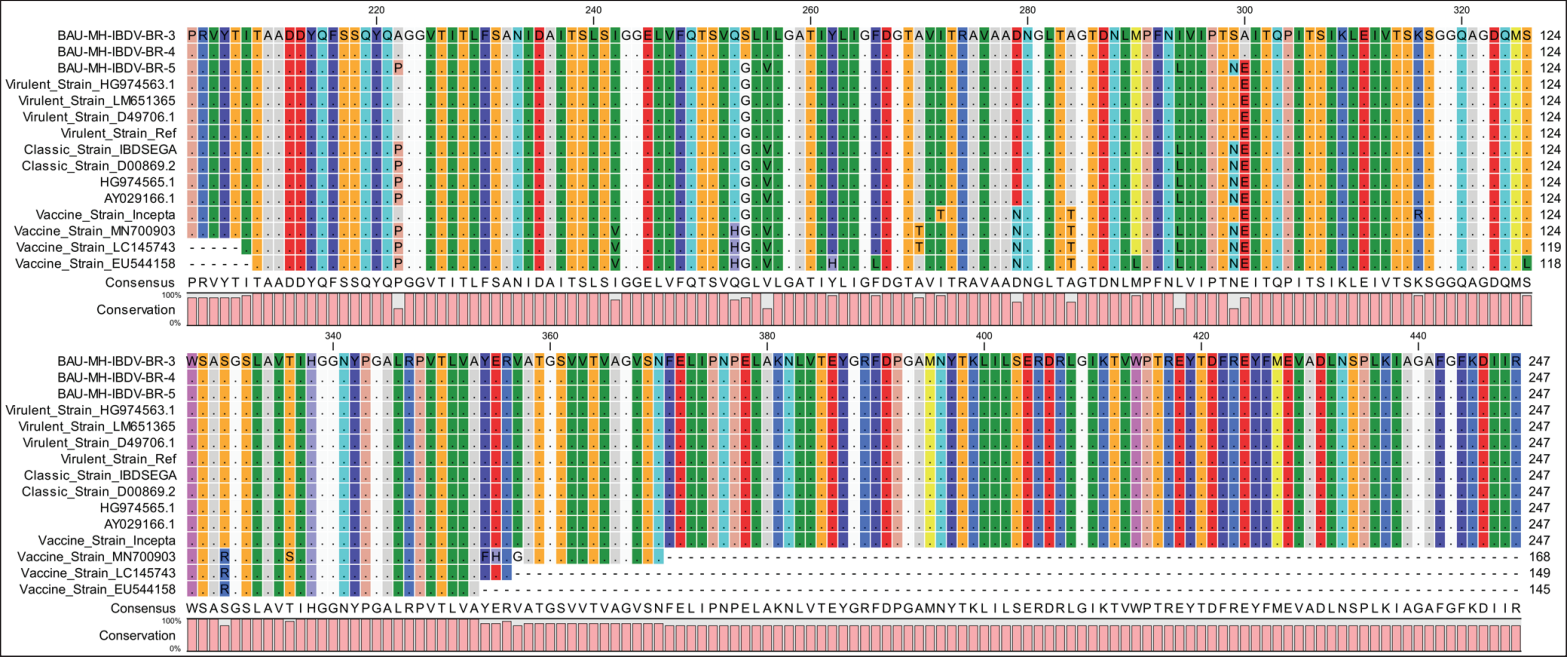
BAU-MH-IBDV-BR-3 and BAU-MH-IBDV-BR-4 strains show more amino acid sequence homology with the vvIBDV strain (GenBank: LM651365.1) with the classic strain and vaccine strain of IBDV.

The amino acid sequence of isolates from Broiler was deduced from a 743-bp nucleotide sequence that shows significant sequence homology to vvIBDV compared to cvIBDV and the vaccine strain (Fig. 2), with substantial mutations at S254G and E300A. The virus isolated from backyard chickens shows sequence homology with the classic strain and the vaccine strain. Essential amino acids for the virulent marker (253Q and 284A) in the IBDV are present in the sequenced strains isolated from the broiler. HVR with amino acid motifs at A222, I242, I256, I294, and S299 presumed to be attributed to the virulence character of the virus were identified in all four broiler isolates. Whereas isolates from backyard chickens show amino acid residues as classic strains and vaccine strains at P222, V256, L294, and N299.

Amino acid differences between virulent and classic or vaccine strains are illustrated in Figure 2, which are compared with crystal-structured derived VP2 protein (Protein id-2DF7) along with isolated viruses. The four sequenced viruses from broiler and a Chinese virulent strain (Accession No. LM651365) show amino acid sequence homology with the classic strain at I242, D279, and A284 and with the virulent markers at Q253, A284. The vaccine strain shows amino acid changes compared with vvIBDV and the isolated strain at P222A, V242I, H253Q, V256I, H262Y (Vaccine strain Cevac only), L266F (vaccine strain Cevac only), T270A (except Vaccine strain Cevac only), N279D, and L290M (vaccine strain Cevac only). cvIBDV (Accession No. D00869.2) expresses amino acid sequence homology with vvIBDV and four sequenced strains at P222 and V256 amino acids. The amino acid motif at G254S, E300A represents mutation, which is absent in most vvIBDV, classic strains, and vaccine strains.

**Table 1.** Isolated IBDV GenBank accession numbers and host.

Isolate name	Accession numbers	Host	Strain
BAU-MH-IBDV-BR-1	MT090078	Broiler	vvIBDV
BAU-MH-IBDV-BR-2	MT090079	Broiler	vvIBDV
BAU-MH-IBDV-BR-3	OR361705	Broiler	vvIBDV
BAU-MH-IBDV-BR-4	OR361706	Broiler	vvIBVD
BAU-MH-IBDV-BR-5	OR361707	Back yard chicken	cvIBVD
BAU-MH-IBDV-BR-6	OR361708	Back yard chicken	cvIBVD

**Figure 3.**
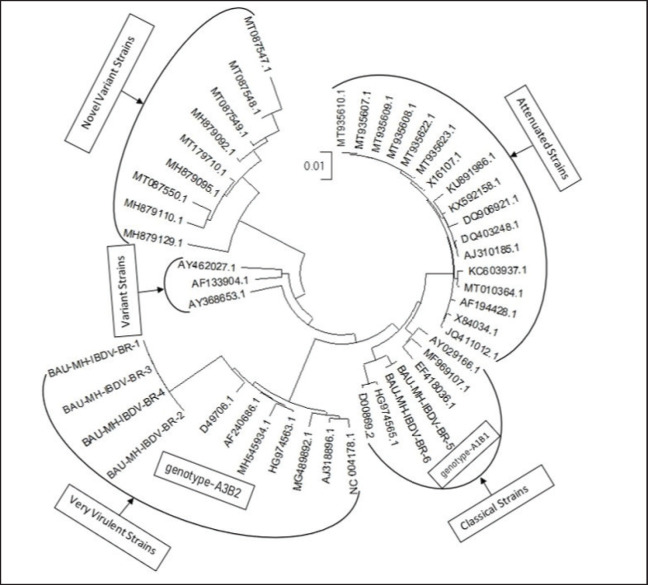
The evolutionary history was inferred using the maximum likelihood method and the Tamura-Nei model.

### IBDV isolation in SPF eggs

**Figure 4.**
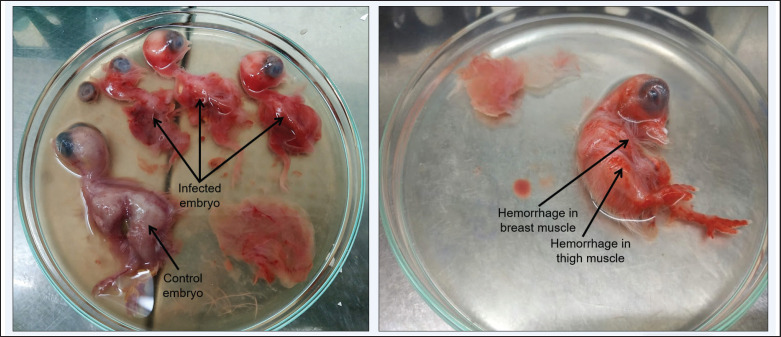
Hemorrhage all over the embryo body including in breast and thigh muscles after inoculation of processed raw field samples.

Inoculation of samples from Broiler showed a high embryo mortality rate of 100% after 36–48 h of inoculation, and all the embryos died within 45–50 h of inoculation. In the fourth passage, embryonic mortality was reduced to 95%. On the other hand, inoculation of samples collected from backyard chickens showed embryo mortality of 95% after 65–80 h of inoculation. And on the fourth passage, embryonic mortality dropped to 65%. Embryos were found to have massive hemorrhage in the embryo body, including in breast muscle and thigh muscle, thickening of CAM, and swelling in the kidneys (Fig. 4). The embryonic body and CAM were collected, and the virus was detected by RT-PCR.

### Pathogenicity test results

**Figure 5.**
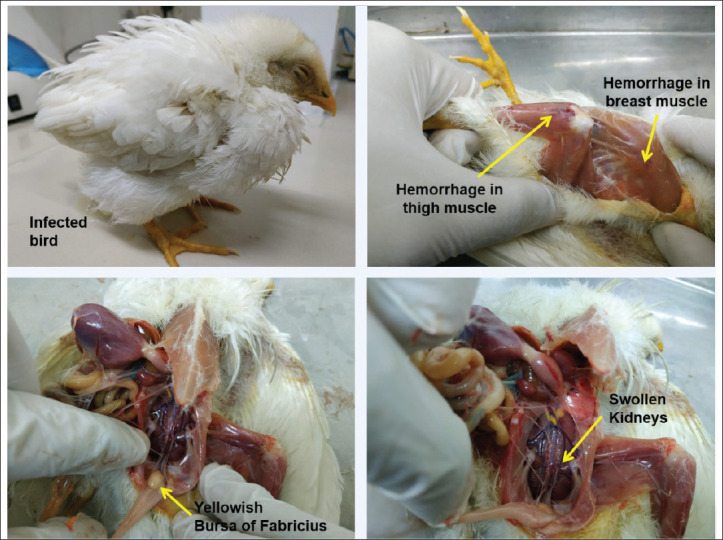
Hemorrhage in breast and thigh muscle, swelling of the kidney, and yellowish enlarged BF were evident in the pathogenicity test of the virus in seronegative chicken.

**Figure 6.**
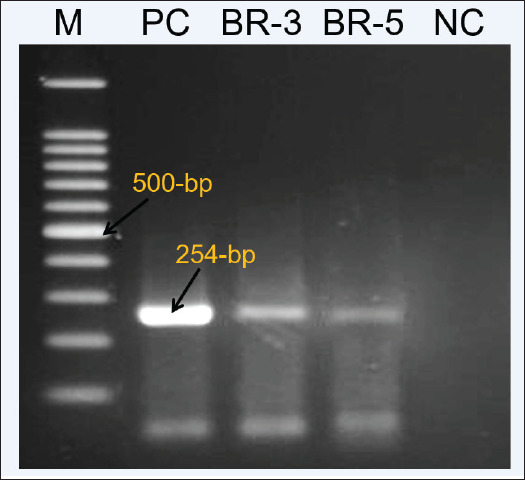
IBD virus detected in infected chicken after pathogenicity test following RT-PCR.

Pathogenicity in seronegative chickens, Chickens in the BR-3 test groups started showing symptoms of IBD after 3 days of virus inoculation, whereas chickens in the BR-5 group showed mild to no symptoms of the viral infection. Major symptoms of the virus infection in the BR-3 test group showed raffled father, tumbling movement, white watery diarrhea, anorexia, depression, and dehydration. No deaths of chicken occurred for both the BR-3 and BR-5 test groups. Birds were subjected to postmortem observation for clinical signs of IBD. In the BR-3 test group, infection in chickens is characterized by hemorrhage in the thigh and breast muscles (Fig. 5). Distinctive enlarged yellowish BF and swelling of the kidney were present in the chickens of the BR-3 and BR-5 groups compared to the control group (Fig. 5). IBDV was detected in the tissue by RT-PCR (Fig. 6).

**Figure 7.**
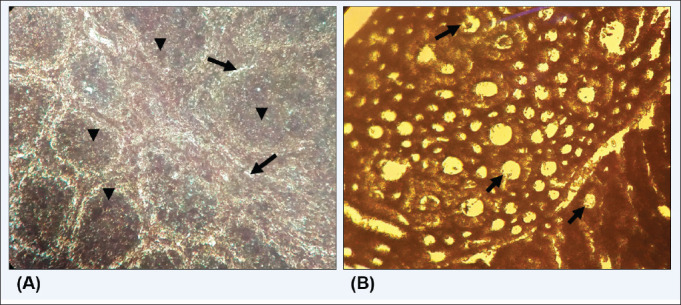
Histopathological changes with vvIBDV infection in chicken organs. A. Kidney, B. Bursa of Fabricius.

In histopathological examination, lesions were observed in the muscle and breast tissue of the BR-3-infected chicken. Histopathological lesions were also evident in the kidney and BF tissue of BR-3 and BR-5 test chickens (Fig. 7). Moreover, virulent isolates successfully developed infection with visible signs of the disease, which were evident in post-mortem lesions. On the other hand, infection with the classic strain (BR-5) showed mild symptoms, but the infection was apparent in the BF.

## Discussion

In this study, the isolation source of IBDV involved backyard chickens and vaccinated broilers in Bangladesh. Out of the 77 field samples tested, 33 of them (equivalent to 42.85%) showed a CPE on CF cells. Meanwhile, the virus was detected in 24 samples (equivalent to 31.16%) through molecular RT-PCR. Therefore, RT-PCR is an effective method to detect IBDV in tissue samples [18].

The virus isolated from broiler chicken showed a higher virulence compared to the virus isolated from backyard chicken when SPF eggs were injected with a processed sample. High embryo mortality was observed before, and embryo mortality remains higher after the fourth passage for broiler isolates [19]. Whereas for backyard chicken-isolated IBDV embryos, mortality dropped in the fourth passage to less than 60% for backyard isolates [20]. Identical lesions were evident, including massive hemorrhage in the cerebrum, breast, and thigh muscles, enlargement and congestion in the kidney, and thickening of CAM [19,20].

Pathogenicity in seronegative chickens: the BR-3 group expressed signs and symptoms of IBD within 3–5 days of infection [20,21]. Postmortem lesions of these infected chickens include hemorrhage in the breast and thigh muscles with swollen kidneys and an enlarged BF, which was previously reported [20–22]. On the other hand, chickens in the BR-5 group showed mild to no symptoms of IBD, and postmortem lesions included enlargement of the BF and swelling of the kidney with less to no hemorrhagic lesions in the breast and thigh muscles. However, in phylogenetic analysis, BR-1, BR-2, BR-3, and BR-4 viruses clustered with vvIBDV, which belongs to genotype A3B2. BR-5 and BR-6 clustered with the classic strain of the IBDV virus, which belongs to genotype-A1B1. In the histopathology examination, severe lesions were evident in the kidney and BF for infection with the BR-3 virus. Depletion of lymphocyte cells was observed for both BR-3 and BR-5 virus infections [23].

Compared to vvIBDV isolated from broilers (BR-1, BR-2, BR-3, and BR-4), viruses isolated from backyard chickens show sequence homology with cvIBDV and vaccine strains. Though some of the key amino acid residues at I242, D279, and A284 and the virulent marker at Q253, A284 are in BR-5, the BR-6 strain shares the same amino acids as vvIBDV, which may act on the virulence of the virus [21].

Deduced amino acid sequences from four partially sequenced strains (isolated from broiler) were compared with vvIBDV, cvIBDV, and the vaccine strain (Fig. 2). Analyzing the HVR sequence of the isolated virus (BR-1, BR-2, BR-3, and BR-4), amino acids in positions A222, I242, I256, I294, and S299 represent putative markers for pathogenic IBDV [14,24], four of which are absent in the virus isolated from backyard chicken. Amino acid residues 253Q, 279D, and 284A are present in both cvIBDV and vvIBDV, which are mutated in vaccine strains at 253H, 279N, and 284T amino acids because of cell adaptation or attenuation processes [25,26]. Although routine vaccinations are practiced on farms, frequent antigenic changes in the virus may result in vaccination failure or the breakdown of maternal antibodies [1,27]. Amino acid G254 in vvIBDV is mutated in BR-1, BR-2, BR-3, and BR-4 strains with the S254 residue, which is absent in most IBDV viruses. However, the presence of amino acids at S254 was also seen in other virulent IBD virus strains (Accession No. AZI15612.1, MH137949.1), which were identified in juvenile chickens vaccinated with the classic vaccine type of the virus [8,28]. However, such mutations may have an impact on the breakthrough of immunity by maternal antibodies or different classical vaccine strains.

The loss of pathogenicity of the virus has been observed as a result of substitution of amino acid motifs at Q253 with H253, D279 with N279, and A284 with T284 within the VP2 domain of vvIBDV, and interestingly, the introduction of a single point mutation at H253 with Q253 or N253 and R249 with Q249 significantly enhanced the virulent nature of an attenuated IBDV strain [29]. Substitution of amino acids at E300A was reported to cause antigenic drift, and in the G254 with S254 (BR-1, BR-2, BR-3, and BR-4), which may have a potential impact on the virulence of IBDV, glycine replacement with serine at location 254 was reported to cause vaccination failure [27,29]. Furthermore, S254 and 253 Q amino acid positions were also found in a variant strain that was first reported in the USA, and Q253 was involved in the *in vivo* infection [25,30]. However, BR-1, BR-2, BR-3, and BR-4 share genetic elements common to both virulent strains depicted in Figure 2. The virulence of the virus is influenced by mutations in the HVP2 region, whether they occur individually or in combination, although segments A and B both have an impact on the virulence of the IBD virus [12,13]. From the previous data, genetic changes in the IBDV genome in HVR of VP2 were reported to allow the virus to escape host defense and expression variations in antigenic characteristics, cell tropism, virulence, and pathogenic phenotype [8,21,25,27].

The present study revealed that mutations in the G254–S254 amino acid motif in the vvIBDV virus circulating in broiler chickens in Bangladesh might cause vaccination failure in broiler chickens. Moreover, from the study, it is exposed that vaccination of broiler chickens with a classical vaccine strain cannot protect against the newly emerged vvIBDV virus. To prevent an outbreak, the development of a vaccine strain should be carried out from a classical virulent strain or a vvIBDV strain.

## Conclusion

Continuous surveillance of emerging IBDV isolates is crucial to studying their antigenic characteristics and assessing their potential to overcome host immunity conferred by introducing current vaccines. During the acute stage of IBD, clinical manifestations can hamper the effectiveness of vaccinations in broiler chickens. A similar study in layer and Sonali chickens should be carried out to identify the novel IBDV to prevent vaccination failure. Thus, frequent mutations of the virus can emerge as novel viruses that should be routinely investigated and characterized to design new vaccines. Such efficient steps can prevent the outbreak of the virus, and monoclonal antibodies can be introduced against the wild virus for rapid treatment, which can efficiently reduce the spread of the virus.

## List of Abbreviations

bp, Base pair; BR, BAU-MH-IBDV-BR; BAU-MH-IBDV-BR-6; BF, Bursa of fabricius; CAM, chorioallantoic membrane;; cvIBDV; Classic virulent infectious bursal disease virus; cDNA, Complementary deoxyribonucleic acid; CPE, Cytopathic effect; HVR, hypervariable region; IBD, Infectious bursal disease; PBS, Phosphate-buffered saline; SPF, Specific pathogen Free; vvIBDV, Very virulent infectious bursal disease virus. °C, Degree Celsius
